# Flavor Characteristics of Sun-Dried Green Tea in Different Regions of Yunnan: Metabolite Basis and Soil Influencing Factors

**DOI:** 10.3390/foods14071280

**Published:** 2025-04-07

**Authors:** Miao Zhou, Xiujuan Deng, Qiaomei Wang, Zhenzhen Wei, Xinhua Wang, Wenxia Yuan, Limei Li, Man Zou, Weihao Liu, Shijie Lu, Yubo Sheng, Baijuan Wang

**Affiliations:** 1 College of Tea Science, Yunnan Agricultural University, Kunming 650201, China; 18724979985@163.com (M.Z.); xiujuandeng@foxmail.com (X.D.); wqm19850127@163.com (Q.W.); wangaoyu2011@163.com (X.W.); yuanwenxia2023@163.com (W.Y.); 18214178910@163.com (L.L.); 15770383235@163.com (M.Z.); liuweihao2210@163.com (W.L.); 18087238940@163.com (S.L.); 2 China Tea (Yunnan) Co., Ltd., Kunming 650217, China; weizhenzhen@cofco.com

**Keywords:** sun-dried green tea, flavor characteristic, different regions, tea garden soil

## Abstract

To elucidate the regional flavor characteristics of sun-dried green tea (SDT) and their underlying influencing factors, a comprehensive analysis was conducted using metabolomics and flavoromics approaches. This study systematically examined SDT samples and their corresponding tea garden soils from 13 distinct regions in Yunnan Province. The results revealed that the SDT samples could be classified into two distinct groups based on their flavor profiles. Compared to the regions of Pa Sha (PS), Bang Dong (BD), Dong Ban Shan (DBS), Dong Guo (DG), Su Hu (SH), Gua Feng Zhai (GFZ), and Wu Liang Shan (WLS), the regions of Xin Nong (XN), Ba Ka Nuan (BKN), Mang Ang (MA), Man Nuan (MN), Bing Dao (BDao), and Bin Shan (BS) exhibited a significant upregulation of the tea polyphenols (TP)/free amino acids (FAA) ratio. The former group was characterized by a sweet mellow taste, while the latter displayed a stronger taste profile. Furthermore, the analysis of volatile compounds demonstrated that geraniol and linalool were significantly upregulated in the PS, BD, DBS, DG, BS, and BDao regions, which were associated with tender and floral aromas. In contrast, isophorone, 2-pentyl furan, 1-octanol, D-limonene, and benzaldehyde were markedly enriched in the XN, BKN, MA, MN, SH, GFZ, and WLS regions, contributing to sweet and honey-like aromatic profiles. Altitude and mineral element phosphorus are potential key factors affecting the regional flavor differences in SDT. Specifically, SDT cultivated at higher altitudes and in soils with elevated available phosphorus content exhibited a greater likelihood of accumulating sweet mellow and floral compounds. This study provides scientific evidence for understanding the characteristic flavor profiles of SDT across different regions, offering valuable insights into the factors contributing to regional flavor differentiation in tea production.

## 1. Introduction

Yunnan’s favorable geography and climate have given rise to Pu-erh tea [1]. In recent years, Pu-erh tea has become increasingly popular with consumers because of its unique health benefits and distinctive flavor [2]. As the foundation of Pu-erh tea’s characteristic flavor, the Yunnan large-leaf variety of SDT plays a crucial role in determining the final product quality. Therefore, comprehensive research on the terroir-specific quality attributes of the Yunnan large-leaf variety of SDT is essential for establishing effective quality control measures throughout Pu-erh tea production processes. This investigation is particularly critical for maintaining the unique regional characteristics and ensuring consistent quality in Pu-erh tea manufacturing.

Volatile organic compounds (VOCs) constitute essential components that significantly contribute to the complex flavor profile of SDT. Extensive research has identified alcohols, ketones, and hydrocarbons as the predominant classes of volatile compounds in SDT [3,4]. The samples from each producing area had their own characteristic volatile components [5], such as different townships in Menghai County, where the SDT aroma is dominated by fruity, floral, and woody flavor profiles [6]. Advanced analytical techniques, including headspace solid-phase microextraction-gas chromatography-mass spectrometry (HS-SPME-GC-MS), gas chromatography-ion mobility spectrometry (GC-IMS), and electronic nose analysis, have proven effective in discriminating SDT aroma profiles across different Yunnan tea-growing regions [4,5,7]. However, the specific key flavor compounds underlying these regional variations remain to be fully elucidated.

Research on SDT has revealed significant differences in the chemical composition of tea from different production regions. For instance, SDT from Bulang Mountain has the highest water extracts (WE) and TP content, while the FAA content is lower than that of SDT from Nannuo Mountain [8]. Sensory evaluation coupled with biochemical analysis has demonstrated that SDT from the Yiwu tea region consistently surpasses that from the Linxiang region in overall quality metrics [9]. Furthermore, altitudinal influences on SDT composition have been identified, with low-altitude SDT from Wuliang Mountain containing elevated TP levels, contributing to a more pronounced bitter and astringent taste profile [10]. Conversely, high-altitude SDT exhibits increased FAA content, resulting in enhanced freshness and briskness. Seasonal variations have also been observed, with spring tea containing the highest FAA levels, summer tea being rich in total catechins, and autumn tea being characterized by elevated TP content [11].

The environmental conditions surrounding tea cultivation areas, including soil, light, temperature, and altitude, can create unique microclimates [12]. These microclimates contribute to the unique “local flavor” of Yunnan SDT. Soil, as the basis for the survival and growth of the tea tree, along with its physical and chemical properties, and fertility, are the main factors affecting the growth of tea trees and the quality of tea [13]. FAA in tea are precursors for the production of volatile compounds [14], and the accumulation of FAA is affected by the nitrogen content in the soil. Appropriate application of nitrogen fertilizer can promote the formation of amino acid flavor substances, but excessive application of nitrogen fertilizer may lead to an increase in grassy flavor and reduce the aroma of tea [15,16]. In addition, phosphorus and magnesium can improve tea quality by affecting the synthesis of aromatic substances and promoting the Maillard reaction [16]. Phosphorus fertilization increases the content of TP, while potash application increases the total amount of catechins [17,18]. Application of large amounts of phosphorus and potassium in tea gardens promotes malic acid metabolism in tea buds and induces redistribution of photosynthetic products and carbohydrates in favor of the catechin pathway, which further affects tea quality [19]. Differences in SDT quality are closely linked to the environmental conditions around the tea-producing areas; therefore, the key components responsible for these differences require further investigation.

Given the established correlation between SDT quality variations and the environmental conditions of tea-producing regions, further investigation is warranted to elucidate the key components responsible for these qualitative differences. This research direction would provide valuable insights into the complex interactions between environmental factors and tea quality parameters.

## 2. Materials and Methods

### 2.1. Preparation of Tea Samples

The SDT samples in this study came from 13 origins in the Digital Tea Plantation Base established by China Tea (Yunnan) Co. Ltd. (Kunming, China) and Yunnan Agricultural University which were named after the respective townships. Tea samples were collected from five randomly selected sampling points at each tea plantation, with each sampling point having the same depth (rhizosphere, 20 cm). Approximately 1 kg of soil, consisting of a mixture of soil samples, were collected from each sampling point [20]. The specific details of the sample collection sites are provided in Table 1.

### 2.2. Sensory Evaluation

The sensory review refers to GB/T23776-2018 (“Tea sensory review method”) [21]. Among these, the sensory quantitative descriptive analysis method collects sensory primitives related to the aroma and taste of SDT, combines the flavor and quality characteristics of Yunnan large-leaf variety of SDT, and comprehensively formulates the SDT sensory quantitative evaluation table. The aroma evaluation record table has 11 evaluation dimensions: pure and normal, strong and rich, high and upward, lasting, clean and refreshing, flowery aroma, honey aroma, sweet aroma, tender aroma, and grass odor. The taste evaluation record table has 10 evaluation dimensions–strong, thick, mellow, pure, sweet, smooth, sweet aftertaste, fresh and brisk, bitter, and astringent. The quantitative evaluation of sensory characteristics is based on a scale of 0–5 to rate their intensity (0 for none, 5 for the strongest intensity) [22].

### 2.3. Analysis of Nonvolatile Compounds

Referring to GB/T8305-2013 (“Determination of water extracts of tea”) [23], GB/T8314-2013 (“Determination of total free amino acids of tea”) [24], and GB/T8313-2018 (“Detection methods for tea polyphenols and catechins in tea”) [25], the water extracts (WE), total free amino acids (FAA), and tea polyphenols (TP) were determined, respectively. The anthrone sulfuric acid method was used to determine soluble sugars (SS) [26].

The determination of catechin, flavonoid, and purine alkaloid fractions was carried out by high-performance liquid chromatography (HPLC) following the method described by Yang [27]. The instrument used in this study is a 1200 high-speed liquid chromatograph equipped with a C18 column (4.6 mm × 100 mm, 2.7 µm, Agilent (Santa Clara, CA, USA)). This method employed a mobile phase A consisting of 0.261% phosphoric acid and 5% acetonitrile and a mobile phase B of 80% methanol. The elution gradient proceeded as follows: mobile phase B increased linearly from 10% to 45% between 0 and 16 min; from 16 to 22 min, mobile phase B increased linearly from 45% to 65%; mobile phase B was held constant at 65% from 22 to 25.9 min; from 25.9 to 29 min, mobile phase B increased linearly to 100%; and mobile phase B was maintained at 100% from 29 to 30 min. The column temperature was maintained at 35 °C. Each sample was extracted and analyzed in triplicate.

### 2.4. Determination of Volatile Components

Electronic nose determination was performed according to the test method of Wu Shanshan et al. [7]. Each tea sample was repeated 3 times.

Headspace solid-phase microextraction (HS-SPME) combined with gas chromatography-mass spectrometry (GC-MS) was utilized to separate and identify the volatile compounds of sun-dried green tea in different regions of Yunnan. The instruments used in this study include a 7890A-5975C headspace solid-phase microextraction GC-MS (Agilent, USA), a DB-WAX column (30 m × 0.25 mm × 0.25 µm, Agilent, USA), and a 65 µm solid-phase microextraction head (PDMS/DVB, Supelco (Bellefonte, PA, USA)). The headspace solid-phase microextraction process was as follows: 1 g of tea sample was placed in a 20 mL headspace vial. A total of 1 µg of ethyl acetate (decanoic acid ethyl ester) was added as the internal standard, followed by the addition of 6 mL of boiling water. The mouth of the vial was then sealed. The CTC autosampler was set with the following conditions: 60 °C, stabilized for 10 min; 65 µm polydimethylsiloxane/divinylbenzene (PDMS/DVB) extraction head; extraction at 60 °C for 30 min with a rotational speed of 250 rpm. For gas chromatography-mass spectrometry (GC-MS) coupled detection, the inlet port temperature was set to 230 °C and the desorption time was 5 min.

GC conditions were as follows: column, DB-WAX (30 m × 0.25 mm × 0.25 µm); carrier gas, He; oven, 50 °C (5 min) to 230 °C (7 min) at 6 °C/min; column temperature, 50 °C (5 min) to 230 °C (7 min); temperature rising rate, 6 °C/min; split ration, no split; MS conditions, mass spectrometry conditions; ion source, EI; gas interface temperature, 280 °C; ion source temperature, 230 °C; quadrupole temperature, 150 °C. Qualitative and quantitative techniques were employed to identify and measure volatile compounds following the approach outlined by Deng et al. [28,29].

For the calculation of relative odor activity value (ROAV), the component with the highest contribution to the flavor of the sample has a ROAV_max_ of 100.00, while the ROAV values of other components are calculated using Formula (1) [30]:

(1) $${ROAV}_{i}=\frac{C_{i}}{C_{max}}\times \frac{T_{max}}{T_{i}}\times 100$$

where C_i_ is the relative content of each volatile component, %; T_i_ is the sensory threshold of each volatile component, mg/kg; C_max_ is the relative content corresponding to the volatile component that contributes the most to the aroma of the tea sample; and T_max_ is the sensory threshold corresponding to the volatile component that contributes the most to the aroma of the tea sample.

### 2.5. Determination of Soil Nutrient Content

For the soil agrochemical analysis, the soil pH was determined using the glass composite electrode method with a soil-to-water ratio of 1:2.5 (m/m). Soil organic matter (SOM) was measured by the potassium dichromate oxidation-external heating method. Total nitrogen (TN) was analyzed using the Kjeldahl method, and alkaline nitrogen (AN) was determined by the alkaline dissolution diffusion method. Total phosphorus (TP) was quantified by the alkali fusion-molybdenum antimony (AMA) colorimetric method, while available phosphorus (AP) was measured using the molybdenum antimony colorimetric method with HCl-NH4F leachate. Total potassium (TK) was analyzed by the alkali fusion flame photometric method, and available potassium (AK) was determined by the flame photometric method with NH4Ac leachate. Additionally, cation exchange capacity (CEC) was measured using the ammonium acetate exchange method.

According to the national environmental technical conditions for tea origin (NY/T853-2004) [31], the fertility grading standards for tea garden soil and the soil nutrition diagnostic indexes for high-quality, high-efficiency, and high-yield tea gardens [32], the soil nutrients of tea gardens were graded.

### 2.6. Statistical Analysis

Excel 2021 was used to process the raw data, MetaboAnalyst (https://www.metaboanalyst.ca/) was used for orthogonal partial least squares discriminant analysis, and Origin2022 and Chiplot (https://www.chiplot.online/) were used for visual representation of the data.

## 3. Results and Discussion

### 3.1. Sensory Flavor Differences in SDT Across Different Regions

**Figure 1:**
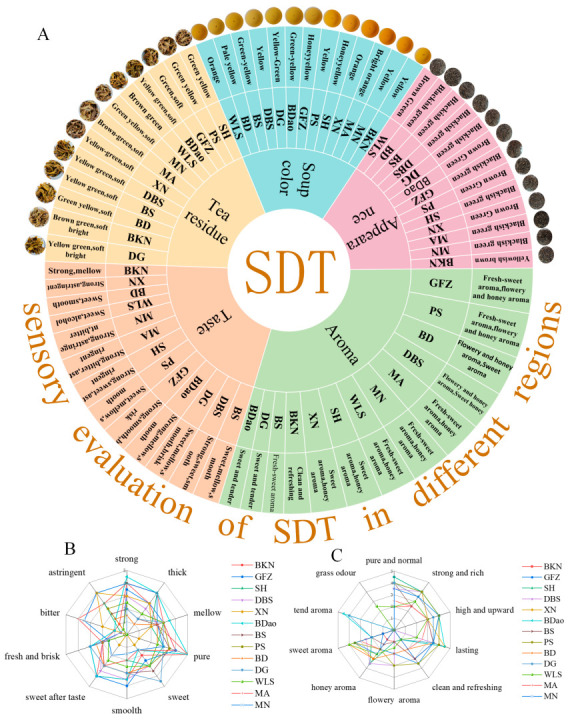
Sensory flavor characteristics of SDT: flavor wheel (**A**); taste radar graph (**B**); aroma radar graph (**C**).

Based on the comprehensive analysis of the review results, a flavor wheel (Figure 1A) and corresponding aroma and taste radar charts (Figure 1B,C) for the Yunnan large-leaf variety of SDT were developed. The analytical results revealed significant regional variations in the color, aroma, and taste profiles of SDT across the 13 investigated regions in Yunnan Province. Regarding chromatic characteristics, the dry tea leaves exhibited a predominant coloration spectrum ranging from dark green to brownish-green, with tightly rolled and substantial leaf morphology. The tea infusion demonstrated a characteristic bright yellow to orange hue, while the brewed leaves displayed a yellowish-green to brownish-green coloration with a soft and lustrous appearance. In the context of tea quality assessment, flavor and aroma profiles emerged as critical evaluation parameters, constituting primary determinants in the comprehensive quality evaluation system of SDT. These sensory attributes were found to play a predominant role in the overall quality assessment framework, underscoring their significance in the organoleptic evaluation of SDT.

In terms of the taste of the tea, the BDao, GFZ, BKN, SH, MA, MN, and XN samples exhibited heavy and thick attributes, of which the BDao, GFZ, and BKN samples had a strong sweet aftertaste, the MA, MN, and XN samples had an obvious or stronger bitterness and astringency, and the SH sample had a more astringent taste. The BS, PS, WLS, BD, DBS, and DG samples exhibited sweet and mellow attributes, with a strong sweet aftertaste. Among these, BS and PS had a sweet, mellow, and slippery taste with some astringency; BD, DBS, and DG had a sweet and smooth taste, with BD and DBS being more mellow and brisk.

In terms of the aroma of the tea, the WLS, MA, and MN samples predominantly exhibited clean sweetness and a honey aroma. The GFZ and PS samples predominantly exhibited clean sweetness, along with floral and honey aromas. The BKN sample predominantly exhibited a clean and refreshing aroma, while SH and XN were mainly characterized by sweet and honey aromas. The BD and DBS samples predominantly exhibited sweet, floral, and honey aromas, while BS had a mainly clean sweetness. The DG and BDao samples predominantly exhibited sweet and tender aromas.

### 3.2. Regional Differences in Non-Volatile Compounds of SDT

The taste properties of tea result from the comprehensive effect of water-soluble substances in the tea infusion on human taste receptors. These are mainly affected by the content and ratio of taste substances, such as polyphenols, alkaloids, amino acids, sugars, and organic acids [33]. Quantitative analysis revealed substantial variation in the concentration ranges of water extract (WE), total polyphenols (TP), free amino acids (FAA), soluble sugars (SS), caffeine (CA), gallic acid (GA), and epigallocatechin gallate (EGCG) across SDT samples from 13 distinct regions. Specifically, the measured ranges were as follows: WE (49.79–52.86%), TP (20.45–26.49%), FAA (1.52–2.87%), SS (4.11–6.10%), CA (36.94–77.12 mg/g), GA (0.48–7.98 mg/g), and EGCG (65.54–85.26 mg/g).

The total polyphenol (TP) content in SDT exhibited significant regional variation across the 13 studied areas, with the MN group demonstrating the highest concentration (26.49 ± 0.27%), followed by the MA group (26.02 ± 0.98%). Conversely, the free amino acid (FAA) content was lowest in the MN group (1.52 ± 0.33%), with the MA group showing the second-lowest concentration (1.67 ± 0.14%). Consequently, the TP/FAA ratio reached its maximum in the MN group (18.09 ± 4.35), followed by the MA group (15.84 ± 1.29).

Samples from GFZ and SH regions were characterized by pronounced bitterness and astringency, accompanied by a distinct heavy and thick mouthfeel. Notably, the TP/FAA ratios in the WLS, PS, DG, DBS, SH, GFZ, and BD groups were consistently below 10, with the GFZ group exhibiting the lowest ratio (7.51 ± 0.90), followed by the SH group (7.82 ± 0.75). The SH group simultaneously displayed the highest FAA content (2.87 ± 0.28%) among all the samples. Interestingly, despite their initial bitterness, the GFZ and SH samples developed a noticeable sweet aftertaste.

The WLS group exhibited unique chemical characteristics, including the lowest flavonoid (FN) content (3.69 ± 0.31%), which correlated with reduced bitterness perception. This group also demonstrated the highest concentrations of total catechins (235.94 ± 0.85 mg/g), gallocatechin gallate (GCG) (18.92 ± 0.48 mg/g), and catechin (CA) (77.12 ± 0.98 mg/g), resulting in a predominantly sweet and mellow flavor profile with a persistent sweet aftertaste. This sensory characteristic may be attributed to the formation of hydrogen bonds between alkaloids and catechins in the tea infusion, with the resulting hydrogen bond complexes potentially enhancing the mellow and fresh characteristics of the tea infusion.

**Figure 2:**
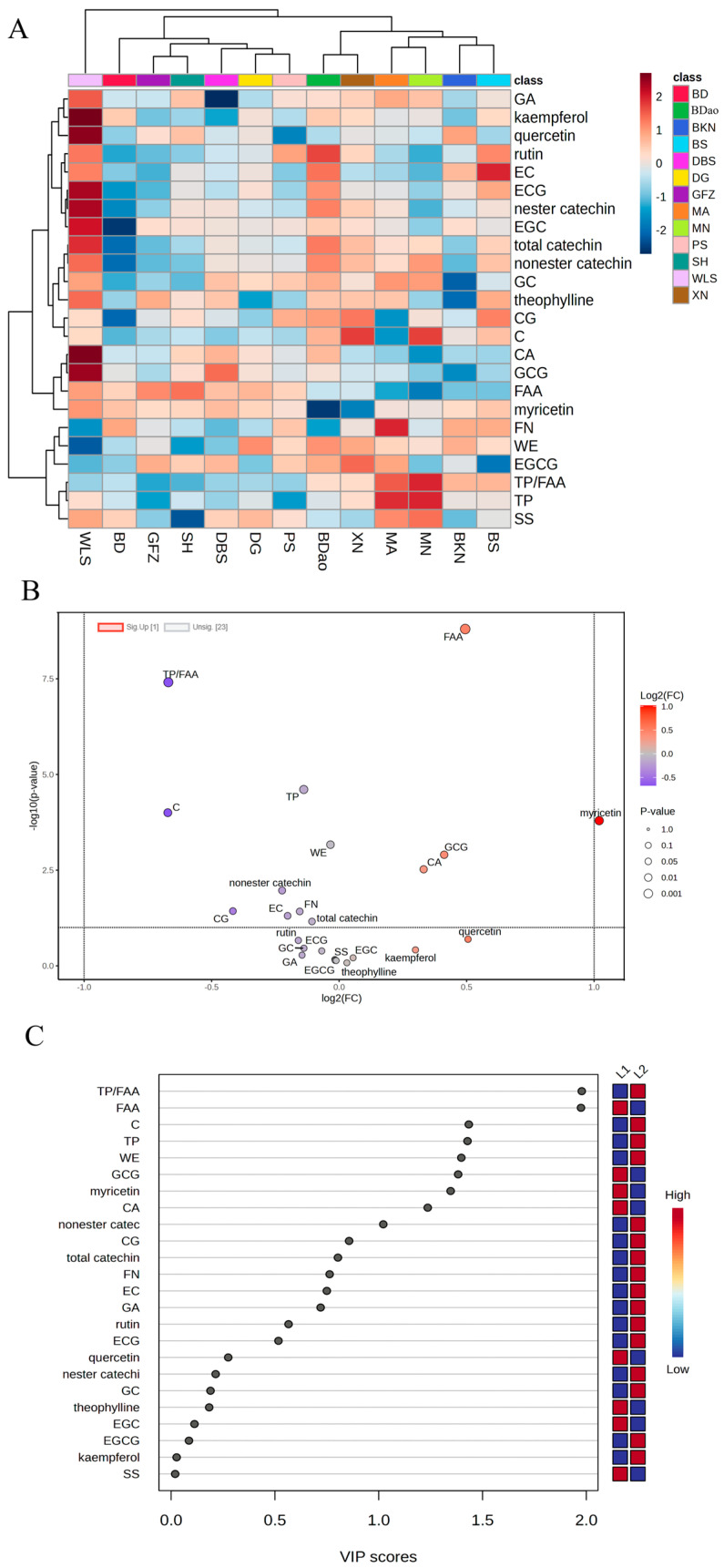
Analysis of regional differences in non-volatile compounds in SDT: heat map (**A**); volcano map (**B**); and VIP value map (**C**).

Cluster analysis revealed that the SDT samples from 13 distinct regions could be categorized into two primary groups based on their chemical composition profiles. The first group (designated as L1) comprised tea samples from the PS, BD, DBS, DG, SH, GFZ, and WLS regions, while the second group (L2) included samples from the XN, BKN, MA, MN, BDao, and BS regions (Figure 2A). Quantitative analysis demonstrated that the TP/FAA (total polyphenols to free amino acids) ratio in L1 group samples was consistently below 10, correlating with a predominantly sweet and mellow flavor profile. In contrast, L2 group samples exhibited TP/FAA ratios exceeding 10, corresponding to a heavier and thicker flavor characteristic.

Orthogonal partial least squares discriminant analysis (OPLS-DA) was subsequently conducted to identify key differential metabolites between the L1 and L2 groups. The model demonstrated satisfactory goodness of fit, with parameter values of R2X:0.745, R2Y:0.931, and Q2:0.892. According to established validation criteria in multivariate analysis, both R2 (coefficient of determination) and Q2 (cross-validated predictive ability) values exceeding the threshold of 0.5 indicate acceptable model performance in both explanatory and predictive capacities. This analysis identified six significant non-volatile metabolites meeting the criteria of fold change (FC) > 1.5 or <0.8, variable importance in projection (VIP) > 1, and *p*-value < 0.05 (Figure 2B,C). Comparative analysis revealed significant upregulation of TP/FAA and catechin (C) content in the L2 group samples relative to the L1 group. Conversely, FAA, myricetin, GCG, and CA were significantly down regulated in L2 samples. These differential metabolites are potentially responsible for the observed regional variations in SDT taste profiles, providing valuable insights into the chemical basis of regional flavor differentiation in sun-dried tea products.

### 3.3. Regional Differences in Volatile Compounds of SDT

**Figure 3:**
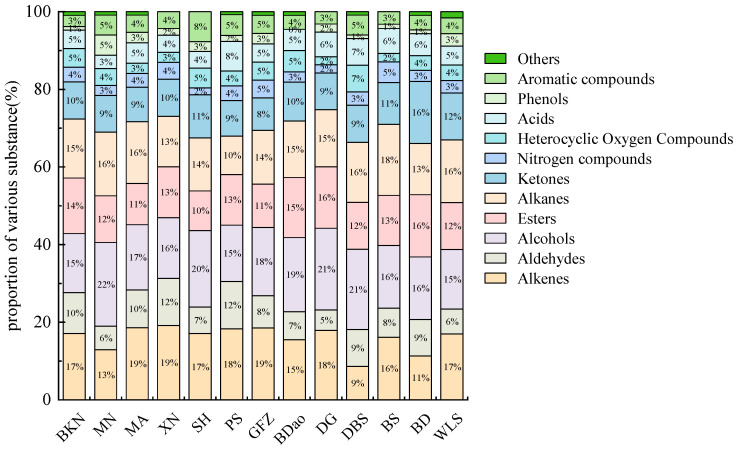
Classification of volatile compounds in SDT.

The aromatic profiles of tea leaves exhibit significant regional variation, playing a pivotal role in determining the flavor quality of SDT [12]. To characterize the volatile organic compounds (VOCs) in SDT, this study employed headspace solid-phase microextraction-gas chromatography-mass spectrometry (HS-SPME-GC-MS). This analytical approach identified 463 distinct volatile compounds, which were systematically classified into 12 chemical categories: 78 alkenes, 66 alcohols, 67 esters, 70 alkanes, 47 ketones, 42 aldehydes, 22 nitrogen-containing compounds, 21 heterocyclic oxygen compounds, 18 carboxylic acids, 10 phenols, 18 aromatic compounds, and 4 miscellaneous compounds (Figure 3). Quantitative analysis revealed substantial variation in volatile composition across different regions. The PS group exhibited the highest diversity of volatile compounds (133 compounds) and the greatest total mass concentration (14.13 mg/g). In contrast, the DG and BS groups demonstrated the lowest compound diversity, each containing 97 volatile compounds. Notably, linalool emerged as the predominant volatile compound across all 13 regional samples, with its relative mass concentration ranging from 0.67 mg/g (XN group) to 26.69 mg/g (DG group).

**Figure 4:**
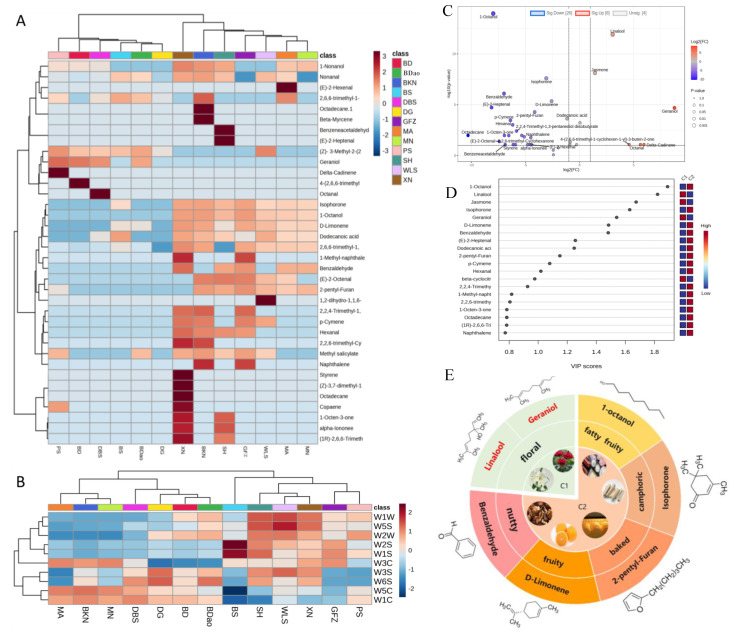
Regional difference analysis of SDT volatile compounds: cluster heat map of 36 aroma active substances (**A**); electronic nose 10 sensor clustering heat map (**B**); volcano chart (**C**); VIP value diagram (**D**); key differential substances (**E**).

Although numerous volatile compounds have been identified in tea, only a limited subset significantly contributes to its sensory profile [34]. These influential volatile compounds, which substantially impact the aromatic characteristics of tea, are scientifically defined as “key aroma substances” [35]. Through comprehensive analysis incorporating aroma threshold values and sensory characterization, 118 aroma-active compounds were identified. Subsequent calculation of their relative odor activity values (ROAV) enabled the selection of 36 key aroma-active compounds with ROAV ≥ 1.00 (Table 2). Cluster analysis of these 36 key aroma-active compounds (Figure 4A) demonstrated distinct regional differentiation, classifying the 13 studied regions into two primary groups. Group C1 comprised PS, BD, DBS, DG, BS, and BDao regions, characterized by predominant tender and floral aroma profiles. Group C2 included XN, BKN, MA, MN, SH, GFZ, and WLS regions, exhibiting distinctive clean-sweet and honey-like aromatic characteristics. This classification pattern exhibited remarkable consistency with the cluster analysis results obtained from electronic nose detection data (Figure 4B).

Orthogonal partial least squares discriminant analysis (OPLS-DA) provides a robust statistical approach for the systematic classification of distinct sample groups. To elucidate the critical volatile compounds contributing to regional differentiation in SDT aroma profiles, OPLS-DA was employed to analyze the volatile organic compounds (VOCs) between C1 and C2 groups. The model demonstrated satisfactory goodness of fit, with parameter values of R2X = 0.726, R2Y = 0.996, and Q2 = 0.993. According to established validation criteria in multivariate analysis, both R2 (coefficient of determination) and Q2 (cross-validated predictive ability) values exceeding the threshold of 0.5 indicate acceptable model performance in both explanatory and predictive capacities. Through this analysis, seven key differential aroma-active compounds were identified based on stringent selection criteria (FC > 1.5 or < 0.8, VIP > 1, and *p*-value < 0.05) (Figure 4C–E).

Comparative analysis revealed significant upregulation of five compounds in the C2 group relative to C1: isophorone (woody), 2-pentyl furan (caramel), 1-octanol (fatty), D-limonene (fruity), and benzaldehyde (fruity). Conversely, two floral compounds, geraniol and linalool, were significantly downregulated in the C2 group. These identified volatile compounds represent the primary chemical determinants underlying the regional differentiation in SDT aroma characteristics across production areas. The aroma and flavor profiles of green teas are largely determined by their processing methods. Sun-dried green tea, characterized by its unique “sunlight” aroma, retains a higher concentration of volatile alcohols such as linalool and geraniol, which contribute to its balanced and complex aroma profile. By employing geranyl pyrophosphate as the precursor substrate, geraniol and linalool were synthesized via the catalysis of geraniol synthase and linalool synthase, respectively [36]. These aroma compounds are particularly advantageous for Pu-erh tea fermentation, as the oxidation of linalool enhances the formation of woody aroma compounds, enriching the sensory qualities of Pu-erh tea [37].

### 3.4. Regional Differences in Soil Nutrients

**Figure 5:**
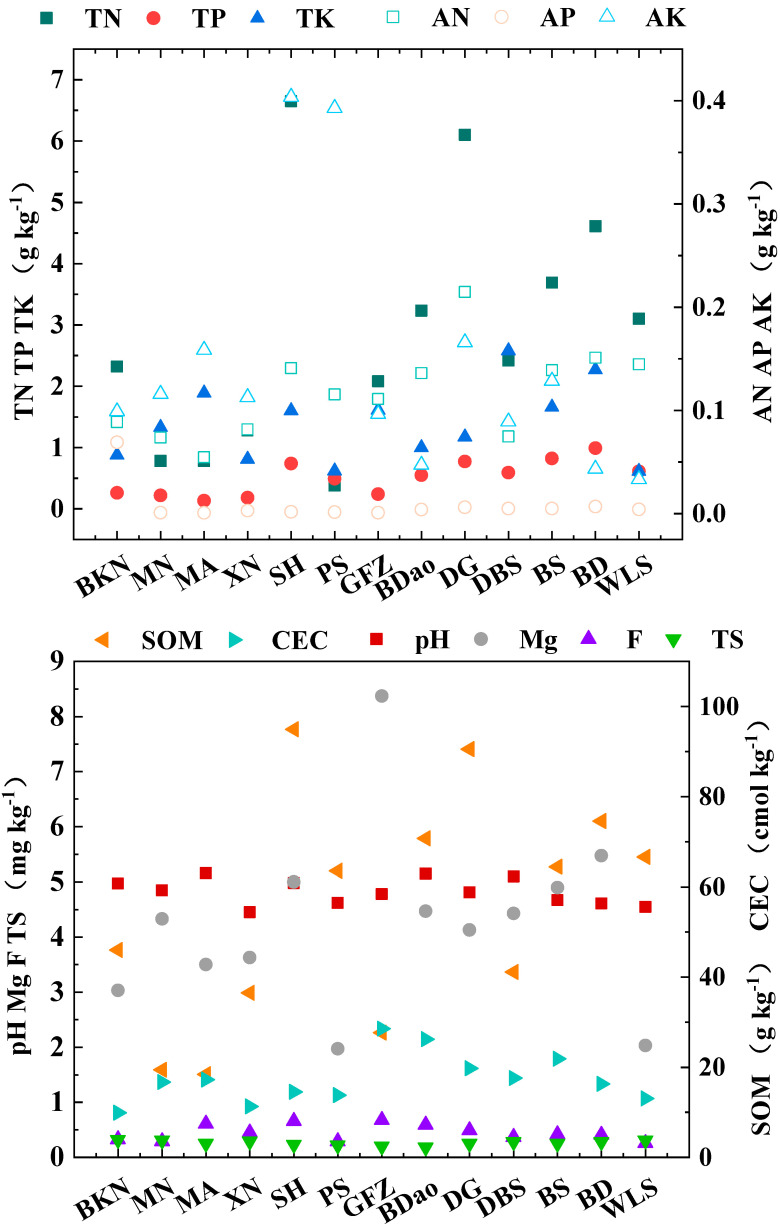
Map of soil nutrient differences.

The soil nutrient analysis results (Figure 5) revealed distinct characteristics across the 13 tea garden regions. The soil pH values ranged from 4.45 to 5.16, with 92.31% of the tea gardens meeting the criteria for high-quality fertility tea gardens, indicating optimal conditions for tea tree growth. Notably, the XN tea garden exhibited a pH of 4.45, which is below the threshold of 4.50, suggesting potential soil acidification issues. Total nitrogen (TN) content varied between 0.78 and 6.10 g/kg, with 84.61% of the tea gardens conforming to high-quality fertility standards. However, the MN and MA tea gardens exhibited TN levels classified as grade III. Available nitrogen (AN) content ranged from 54.67 to 214.67 mg/kg, with 61.54% of the tea gardens meeting high-quality fertility standards. The remaining gardens were distributed as follows: BKN, XN, and BS at grade II, and MN and MA at grade III, indicating generally sufficient soil nitrogen content across all sites. Total phosphorus (TP) and available phosphorus (AP) contents were measured at 0.13–0.77 g/kg and 0.83–6.75 mg/kg, respectively, with both parameters falling within grade II to III levels. Total potassium (TK) content ranged from 0.61 to 2.58 g/kg, with 69.23% of the tea gardens meeting high-quality fertility standards. The remaining gardens BKN, XN, PS, and WLS exhibited TK levels at grade II, accounting for 30.77% of the total. Available potassium (AK) content varied between 33.33 and 403.67 mg/kg, with 61.54% of the tea gardens conforming to high-quality fertility standards. The remaining gardens were distributed as follows: BKN, GFZ, and DBS at grade II, and BDao and BD at grade III. Soil organic matter (SOM) content ranged from 18.4 to 94.9 g/kg, with 84.62% of the tea gardens meeting high-quality fertility standards. The MN and MA tea gardens exhibited SOM levels at grade II, accounting for 15.38% of the total sample.

**Figure 6:**
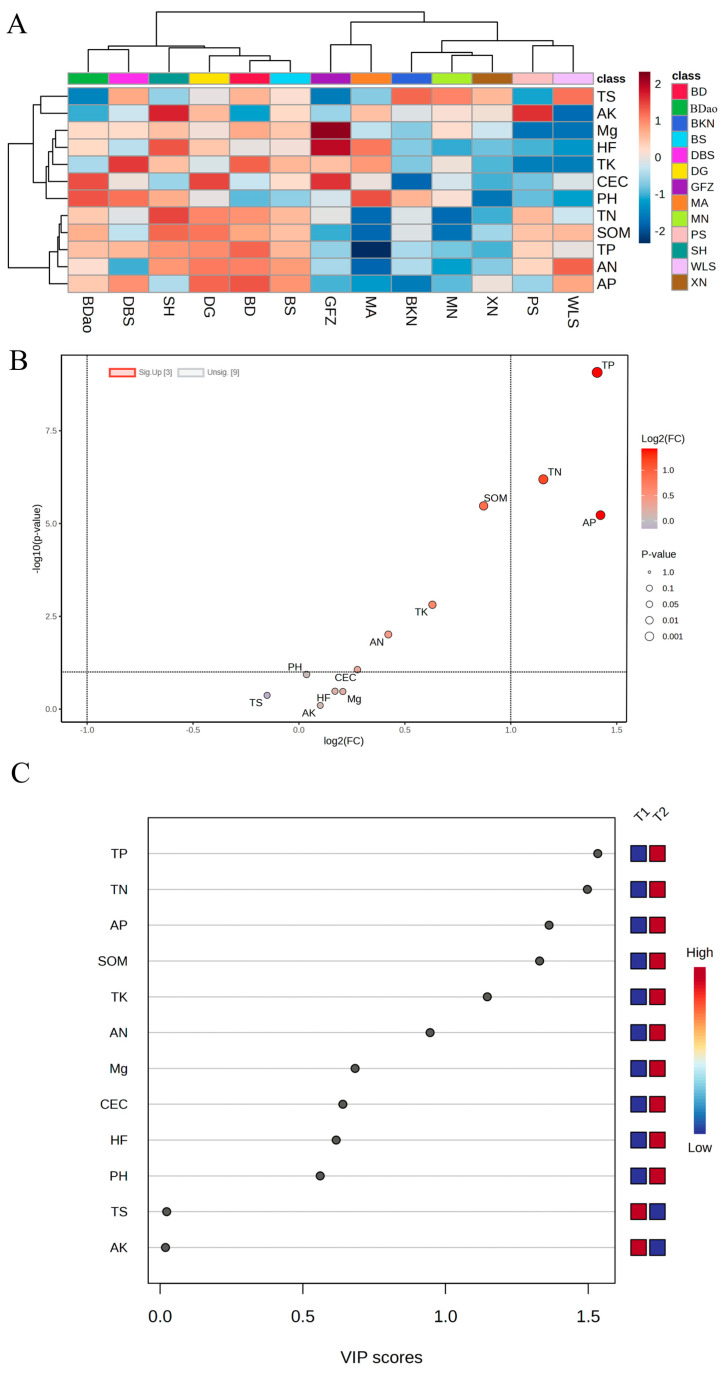
Soil nutrient clustering heat map (**A**), volcano map (**B**), and VIP value map of OPLS-DA (**C**).

Cluster analysis of soil nutrient profiles revealed distinct groupings among the sampling sites. Specifically, SH, BD, DBS, DG, BS, and BDao were classified into the T1 group, while XN, BKN, MA, MN, PS, GFZ, and WLS formed the T2 group (Figure 6A). Statistical analysis demonstrated significantly higher soil AP content in the T1 group (3.94–6.75 mg/kg) compared to the T2 group (0.69–3.00 mg/kg). OPLS-DA was subsequently conducted to identify key differentiating factors between the two groups. The model demonstrated satisfactory goodness of fit, with parameter values of R2X:0.621, R2Y:0.878, and Q2:0.827. According to established validation criteria in multivariate analysis, both R^2^ (coefficient of determination) and Q2 (cross-validated predictive ability) values exceeding the threshold of 0.5 indicate acceptable model performance in both explanatory and predictive capacities. This analysis identified five mineral elements meeting the following criteria: FC > 1.5 or <0.8, VIP > 1, *p* < 0.05. These elements were determined to be the principal mineral components contributing to the intergroup differentiation (Figure 6B,C). Comparative analysis revealed significant upregulation of multiple soil parameters in the T1 group relative to the T2 group, including TP, AP, TN, SOM, and TK. These findings suggest that these soil characteristics may serve as key indicators for the observed grouping patterns and potentially influence the regional differentiation in tea quality parameters.

### 3.5. Factors Affecting SDT Quality Differences Across Regions

The biosynthesis of aroma and flavor compounds in SDT is significantly influenced by regional altitudinal variations and soil nutrient composition. Empirical evidence from previous studies, corroborated by the current investigation, has demonstrated substantial heterogeneity in soil nutrient profiles across different regions of Yunnan Province [13,38]. Furthermore, the enhancement of tea quality has been established as a critical factor in improving the economic value of tea products [39]. To systematically investigate the environmental determinants underlying regional quality variations in SDT, this study employed Pearson correlation analysis and interactive Mantel tests to examine the relationships between soil nutrient content, altitudinal factors, and tea quality components across 13 distinct regions.

**Figure 7:**
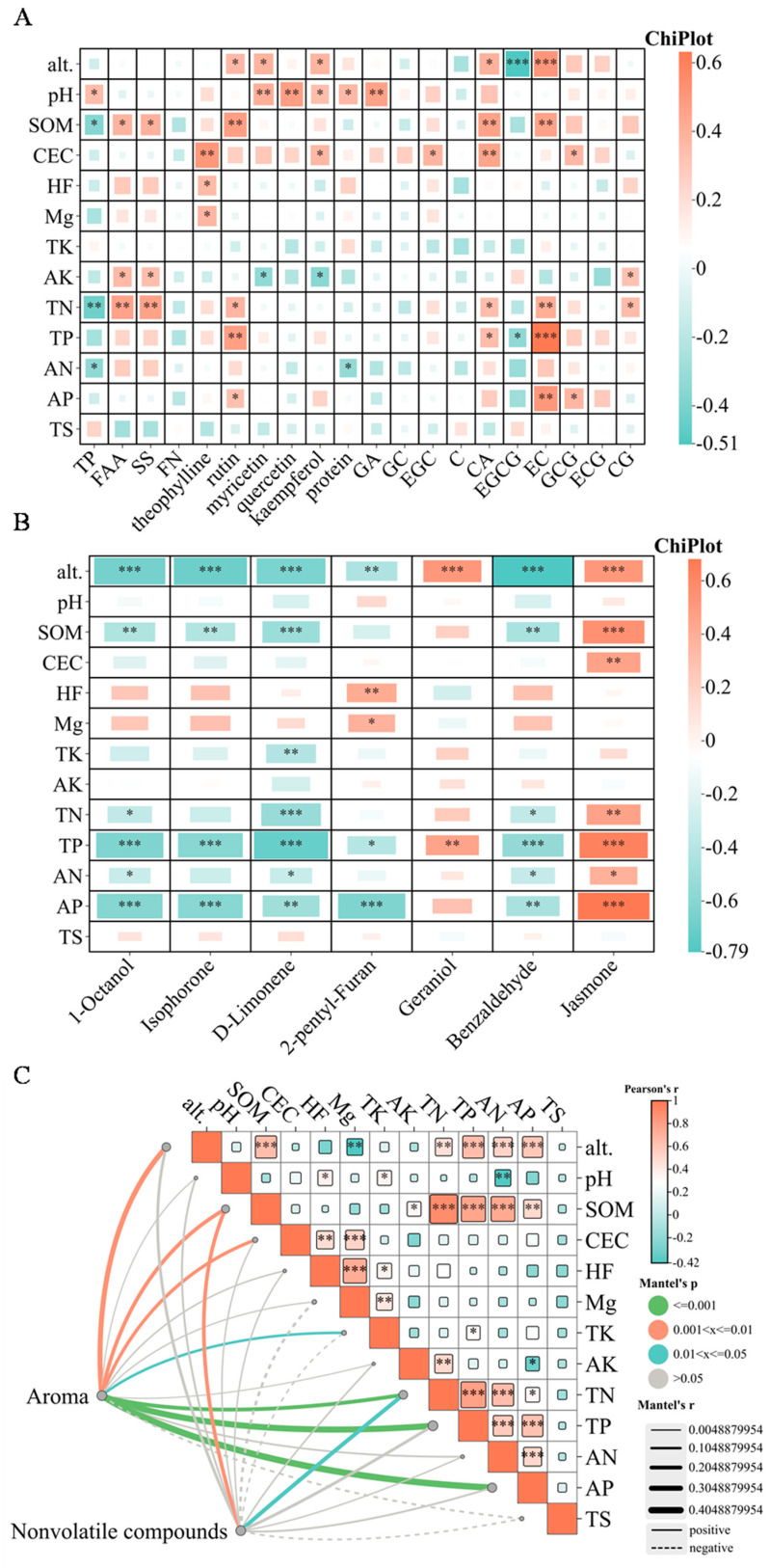
Correlation heat map between environmental factors (**A**,**B**) and interactive Mantel test correlation heat map (**C**). *: *p* < 0.05, **: *p* < 0.01, ***: *p* < 0.001.

The analytical results demonstrated significant positive correlations between soil pH and several biochemical components in SDT, including total polyphenols (TP), myricetin, quercetin, protein content, kaempferol, and GA. Soil parameters, particularly SOM, TN, and AN, exhibited positive correlations with FAA, SS, rutin, EC, and CA in SDT, while showing negative correlations with total polyphenols (TP). Furthermore, SOM, total phosphorus (TP), and AP were positively correlated with rutin, EC, CA, and ECG, but negatively correlated with EGCG (Figure 7A). These findings partially diverge from previous research by Yang [38], which reported positive correlations between SOM, TN, AN and FAA, positive correlations with total polyphenols (TP), and negative correlations with pH and total phosphorus (TP) in Pu’er tea gardens.

Altitude demonstrated significant positive correlations with rutin, myricetin, kaempferol, CA, and EC in SDT, while exhibiting a negative correlation with bitter-tasting EGCG. Analysis of non-volatile compounds revealed distinct taste profiles between sample groups: L1 group samples (TP/FAA < 10) were characterized by a sweet and mellow taste, whereas L2 group samples (TP/FAA > 10) exhibited a heavier and thicker taste profile. Notably, with the exception of GFZ and BDao, L1 group samples were predominantly from higher altitudes compared to L2 group samples, suggesting altitude as a critical environmental factor influencing SDT taste characteristics. Specifically, high-altitude samples tended to demonstrate sweeter and mellower profiles, while low-altitude samples exhibited heavier and thicker taste characteristics.

These variations are potentially attributable to the synergistic effects of light intensity and temperature on tea plant metabolism at different altitudes [12]. The photodegradation of theanine into ethylamine and glutamic acid, which serve as precursors for catechin synthesis [40], combined with reduced light intensity and duration in high-altitude regions due to increased cloud cover and precipitation, may contribute to lower TP/FAA ratios in tea leaves. Given the established role of TP/FAA ratio in determining the balance between heavy/thick and sweet/aftertaste characteristics of tea infusion [41,42], the observed lower TP content and higher theanine levels in L1 group samples likely account for their sweeter and mellower taste profile compared to L2 group samples.

The mineral element phosphorus (P) and altitude emerged as the most significant factors influencing the differentiation in aroma-active substances in SDT. Statistical analysis revealed significant positive correlations between phosphorus content/altitude and floral compounds, specifically geraniol and linalool, while demonstrating negative correlations with isophorone (woody), 2-pentyl furan (caramel), 1-octanol (fatty), D-limonene (fruity), and benzaldehyde (fruity) (Figure 7B). Comparative analysis between groups showed significant upregulation of geraniol and jasmone in C1 relative to C2, whereas isophorone, 2-pentyl-furan, 1-octanol, D-limonene, and benzaldehyde were significantly downregulated.

The clustering analysis of soil nutrients and volatile compounds, excluding the PS and SH regions, demonstrated consistent patterns. The T1 group exhibited higher soil available phosphorus (AP) content compared to T2, while the T1 and C1 groups generally showed higher altitude levels than their T2 and C2 counterparts, with the exception of the WLS region. These findings suggest that soil phosphorus content and altitude serve as potential key markers influencing the floral, woody, caramel, fatty, and fruity characteristics of SDT. Specifically, regions characterized by elevated AP content and higher altitudes demonstrated a greater propensity for the accumulation of floral compounds.

These results align with previous research demonstrating that high-altitude tea samples contain elevated levels of alcohols with floral and fruity characteristics, while low-altitude regions, such as those producing Xinyangmaojian, exhibit a higher proportion of woody compounds [10,43]. The consistency of these findings across different studies reinforces the significance of edaphic and altitudinal factors in shaping the aromatic profile of tea products.

The results of interaction analysis also verified the above conclusions (Figure 7C). This may be due to the long-term growth of tea trees in acidic soil environments. The available phosphorus content in acidic soils is usually at a low level [44]. In order to adapt to this low phosphorus condition, tea trees will promote the synthesis of secondary metabolites, and these secondary metabolites play a positive role in assisting the synthesis of tea flavor substances in the subsequent process. Tea aroma serves as a critical determinant of sensory evaluation and quality attributes. Its formation mechanisms involve stress responses during both pre-harvest natural growth and post-harvest processing of tea plants (*Camellia sinensis*). Stressors enhance the biosynthesis, accumulation, and emission of aroma compounds by activating metabolic pathways, exerting dual functionality: improving tea aroma quality and strengthening stress adaptation through chemical defense mechanisms [45]. Previous studies have shown that the enrichment of phosphorus contributes to the formation of terpenes, aliphatic compounds, and aromatic alcohols such as linalool. Phosphorus can effectively promote the formation of flavonoids in tea and the increase of TP, FAA, and caffeine content, thereby improving the nutritional value and aroma intensity of tea [46,47]; however, the phosphorus content needs to be within a certain range, and high concentrations of phosphorus content are negatively correlated with tea quality [48]. In a related study conducted by Liu et al. [49], in Wuyi Mountain tea plantations, tea plants were subjected to two fertilization treatments: high phosphorus (HP, 112.5 kg P_2_O_5_/ha) and low phosphorus (LP, 60 kg P_2_O_5_/ha). The findings indicated that excessive phosphorus application (112.5 kg P_2_O_5_/ha) significantly reduced the accumulation of key aroma metabolites, such as 3-octen-2-ol, 4,8-dimethyl-1,7-nonadien-4-ol, and 3-ethyl-4-methylpyrrole-2,5-dione, in the high-mountain Shuixian tea variety, highlighting the importance of balanced phosphorus management for optimizing tea quality. The aroma characteristics of SDT ‘local flavor’ can be regulated to a certain extent by regulating the application amount of phosphate fertilizer.

## 4. Conclusions

This study revealed significant regional variations in the quality of SDT. Cluster analysis of non-volatile compounds categorized the samples from 13 regions into two distinct groups—L1 (SH, GFZ, WLS, BD, DBS, DG, and PS) and L2 (BDao, XN, MA, MN, BKN, and BS)—characterized by sweet mellow and strong taste profiles, respectively. Notably, the TP/FAA ratio was significantly upregulated in the L2 group compared to the L1 group. Similarly, cluster analysis of key volatile aroma-active compounds classified the samples into C1 (PS, BD, DBS, BS, BDao, and DG) and C2 (XN, BKN, SH, GFZ, WLS, MA, and MN) groups. The C1 group exhibited dominant tender and floral aromas, whereas the C2 group was characterized by clean-sweet and honey-like aromas. Comparative analysis demonstrated significant upregulation of floral compounds, specifically geraniol and jasmone, in the C1 group, while the C2 group showed significant downregulation of isophorone (woody), 2-pentyl furan (caramel), 1-octanol (fatty), D-limonene (fruity), and benzaldehyde (fruity).

Correlation analysis identified altitude and soil phosphorus content as key factors influencing regional differences in SDT flavor quality. Altitude and available phosphorus (AP) content exhibited positive correlations with floral compounds, as well as with rutin, myricetin, kaempferol, CA, and EC in SDT. Conversely, negative correlations were observed with woody, caramel, fatty, and fruity characteristics, as well as with the bitter and astringent compound EGCG. SDT cultivated in high-altitude regions with elevated AP content demonstrated enhanced sweet mellow characteristics and greater enrichment of floral aroma compounds.

These findings provide scientific data supporting the study of regional flavor characteristics in Yunnan SDT. Phosphorus content plays a critical role in regulating SDT aroma compounds, although its underlying mechanisms require further investigation. Future research should focus on achieving targeted regulation of SDT’s regional flavor profiles through controlled variable experiments and multi-omics approaches. This study contributes to a deeper understanding of the factors influencing SDT quality and offers a foundation for optimizing cultivation practices enhancing flavor characteristics.

## Figures and Tables

**Table 1:** Information on where the samples were collected.

Number	Origin Number	Region	Altitude	Longitude and Latitude
1	Ba Ka Nuan (BKN)	Banzhang Village, Brown Mountain Township, Menghai County, Xishuangbanna Prefecture, Yunnan Province, China	1650 m	E 100°20′, N 21°40′
2	Man Nuan (MN)	Mananan Village, Brown Mountain Township, Menghai County, Xishuangbanna Prefecture, Yunnan Province, China	1300 m	E 100°16′, N 21°37′
3	Mang Aang (MA)	Menghuang Village, Brown Mountain Township, Menghai County, Xishuangbanna Prefecture, Yunnan Province, China	1600 m	E 100°22′, N 21°35′
4	Xin Nong (XN)	Xinlong Village, Brown Mountain Township, Menghai County, Xishuangbanna Prefecture, Yunnan Province, China	1300 m	E 100°16′, N 21°37′
5	Su Hu (SH)	Suhu Village, Gelanghe Township, Menghai County, Xishuangbanna Prefecture, Yunnan Province, China	1650 m	E 100°45′, N 21°97′
6	Pa Sha (PS)	Pasha Village, Gelanghe Township, Menghai County, Xishuangbanna Prefecture, Yunnan Province, China	1700 m	E 100°45′, N 21°97′
7	Gua Feng Zhai (GFZ)	Mahe Village, Yiwu Township, Mengla County, Xishuangbanna Prefecture, Yunnan Province, China	1200 m	E 101°21′, N 21°51′
8	Bing Dao (BDao)	Bingdao Laozhai, Mengku Town, Shuangjiang County, Lincang City, Yunnan Province, China	1900 m	E 99°90′, N 23°27′
9	Dong Guo (DG)	Dongguo Mengku Township, Shuangjiang County, Lincang City, Yunnan Province, China	1750 m	E 99°90′, N 23°20′
10	Dong Ban Shan (DBS)	Nasjiao Village, Mengku Town, Shuangjiang County, Lincang City, Yunnan Province, China	1800 m	E 100°09′, N 23°90′
11	Bing Shan (BS)	Bingshan Village, Mengku Town, Shuangjiang County, Lincang City, Yunnan Province, China	1700 m	E 99°82′, N 23°65′
12	Bang Dong (BD)	Hexin Village, Bangdong Township, Linxiang District, Lincang City, Yunnan Province, China	2000 m	E 100°21′, N 23°56′
13	Wu Liang Shan (WLS)	Shanchong Village, Jingping Town, Jingdong County, Pu’er City, Yunnan Province, China	1900 m	E 100°62′, N 24°09′

**Table 2:** Key active aroma for taxa C1 and C2.

No.	VM	RI	CAS	Class	Odor Description	OT (ug/g)	RMC (ug/g)		ROAV		FC	*p*	VIP
							C1	C2	C1	C2			
1	1-Octanol	1280	111-87-5	alcohols	pungent	0.0220	-	2.27	-	2.79	0.00	0.00	1.89
2	Linalool	1269	78-70-6	alcohols	floral, lily of the valley or magnolia scent	0.0060	11.90	1.42	100	100	8.35	0.01	1.82
3	Jasmone	1623	488-10-8	ketones	floral, jasmine, and celery seed aroma	0.0003	15.85	5.91	15.85	6.80	2.68	0.21	1.67
4	Isophorone	1139	78-59-1	ketones	Saussurea costus	0.0110	0.18	1.53	-	1.89	0.12	0.01	1.63
5	Geraniol	1537	106-24-1	alcohols	rose, geranium	0.0066	3.95	-	4.29	-	438.53	0.01	1.54
6	D-Limonene	936	5989-27-5	alkenes	Fruity, sweet fruity (lemon, citrus peel)	0.0340	0.47	2.79	1.16	3.44	0.17	0.01	1.48
7	Benzaldehyde	1245	100-52-7	aldehydes	Fruity, sweet	0.0300	-	1.14	-	1.43	0.01	0.01	1.48
8	(E)-2-Heptenal	1066	2548-87-0	aldehydes	methylphenidate (stimulant drug used in treating asthma)	0.0030	-	2.53	-	3.73	0.00	0.06	1.26
9	Dodecanoic acid	2171	143-07-7	acids	fruity	0.0072	1.44	3.19	2.13	3.85	0.45	0.23	1.25
10	2-pentyl furan	978	3777-69-3	heterozoa	caramel	0.0060	0.19	3.39	1.05	4.37	0.06	0.01	1.15
11	p-Cymene	1007	99-87-6	alkanes	solvent, gasoline, citrus	0.0114	-	0.75	-	1.47	0.01	0.08	1.08
12	Hexanal	953	66-25-1	aldehydes	Fresh and fruity	0.0050	-	0.65	-	1.31	0.01	0.08	1.02
13	β-cyclocitral	1106	432-25-7	aldehydes	Fruity (almond, mango), light, sweet, floral (rose)	0.0050	4.17	4.05	4.34	15.00	1.03	0.98	0.98
14	2,2,4-Trimethyl-1,3-pentanediol diisobutyrate	1560	6846-50-0	esters	-	0.0140	-	0.51	-	1.36	0.02	0.18	0.93
15	1-Methyl-naphthalene	1567	90-12-0	aromatic compounds	Camphoraceous notes, aged; stimulating	0.0080	-	0.36	-	1.10	0.02	0.18	0.82
16	2,2,6-trimethyl-Cyclohexanone	1052	2408-37-9	ketones	Honey scented, lemon scented	0.1000	-	0.31	-	1.24	0.03	0.33	0.81
17	1-Octen-3-one	1045	4312-99-6	ketones	Metallic Aroma	0.0050	-	1.12	-	1.85	0.01	0.20	0.78
18	Octadecane	1754	127-41-3	ketones	floral (violet), woody	0.0016	-	11.42	-	12.16	0.00	0.19	0.78
19	(1R)-2,6,6-Trimethylbicyclo[3.1.1]hept-2-ene	1325	7785-70-8	alkenes	sandalwood	0.0053	-	0.84	-	1.58	0.01	0.24	0.78
20	Naphthalene	1434	91-20-3	aromatic compounds	Tar, camphor breath	0.0500	-	0.21	-	1.15	0.04	0.34	0.77
21	Octanal	1022	124-13-0	aldehydes	fat, soap, lemon, green	0.0007	0.44	-	1.314	-	49.27	0.30	0.68
22	(E)-2-Octenal	1162	593-45-3	alkanes	Chemical odors	0.0200	-	1.54	-	2.46	0.01	0.38	0.66
23	4-(2,6,6-trimethyl-1-cyclohexen-1-yl)-3-buten-2-one	1618	14901-07-6	ketones	floral	0.0070	0.22	-	1.08	-	24.02	0.30	0.64
24	Delta-Cadinene	1446	483-76-1	alkenes	Medicinal and woody	0.0015	0.56	-	1.42	-	62.04	0.30	0.63
25	Beta-Myrcene	918	123-35-3	alkenes	fruity	0.0150	-	0.011	-	1.07	0.83	0.38	0.59
26	Methyl salicylate	1466	119-36-8	esters	floral, Fruity	0.0400	0.56	1.02	1.25	1.65	0.55	0.40	0.56
27	(E)-2-Hexenal	975	6728-26-3	aldehydes	green, leaf	0.0031	-	0.21	-	1.09	0.04	0.38	0.56
28	Nonanal	1115	124-19-6	aldehydes	Sweet rose-like floral scent	0.0035	6.83	12.75	6.83	18.68	0.54	0.34	0.56
29	Styrene	1001	100-42-5	alkenes	balsamic, gasoline	0.0036	-	0.55	-	1.44	0.02	0.38	0.56
30	(Z)-3,7-dimethyl-1,3,6-Octatriene	977	3338-55-4	alkenes	floral, Herbal	0.0340	-	0.19	-	1.08	0.05	0.38	0.56
31	Benzeneacetaldehyde	1350	122-78-1	aldehydes	Fresh and sweet	0.0063	-	1.06	-	1.94	0.01	0.38	0.50
32	alpha-Ionone	1537	18829-55-5	aldehydes	pungent	0.0130	-	0.254	-	1.14	0.04	0.38	0.50
33	1,2-dihydro-1,1,6-trimethyl-Naphthalene	1441	30364-38-6	aromatic compounds	Fruity (citrus, lemon)	0.0025	-	0.63	-	1.52	0.01	0.38	0.45
34	1-Nonanol	1370	143-08-8	alcohols	Fresh, floral, fatty.	0.0053	0.66	2.58	1.18	3.76	0.26	0.15	0.42
35	Safranal	1356	116-26-7	aldehydes	Saffron, Herbal Woody Fragrance	0.0030	2.46	13.23	2.64	16.86	0.19	0.07	0.36
36	Copaene	1206	3856-25-5	alkenes	Lemon-scented woody, earthy, and piney aroma	0.0060	0.19	0.98	1.05	1.87	0.19	0.47	0.09

- Not identified. VM, volatile metabolites; RI, retention index; class, chemical classification originates from (https://www.chemsrc.com/casindex/, accessed on 7 June 2024); odor description, odor description found in the literature with the database (https://www.flavornet.org/flavornet.html, accessed on 6 January 2025; https://www.thegoodscentscompany.com/search2.html, accessed on 7 January 2025); OT, odor thresholds in water; threshold query using https://www.vcf-online.nl/VcfHome.cfm (accessed on 8 June 2024), References: [6,12,22,28,29,34,35]; RMC, relative mass concentration; ROAV, relative odor activity value; FC, fold change; VIP, variable importance in projection.

## Data Availability

The original contributions presented in the study are included in the article, further inquiries can be directed to the corresponding authors.
